# Phylogenetic relationships of closely-related phlebotomine sand flies (Diptera: Psychodidae) of Nyssomyia genus and Lutzomyia subgenus

**DOI:** 10.1590/0074-02760200220

**Published:** 2020-09-14

**Authors:** Sofía Lorián Moya, Angélica Pech-May, María Gabriela Quintana, Mariana Manteca-Acosta, Oscar Daniel Salomón

**Affiliations:** 1 Ministerio de Salud de la Nación, Administración Nacional de Laboratorios e Institutos de Salud Dr Carlos G Malbrán-ANLIS, Instituto Nacional de Medicina Tropical, Puerto Iguazú, Misiones, Argentina; 2 Consejo Nacional de Investigaciones Científicas y Técnicas, Buenos Aires, Argentina; 3 Universidad Nacional de Tucumán, Instituto Superior de Entomología, San Miguel de Tucumán, Argentina; 4 Ministerio de Salud de la Nación, Administración Nacional de Laboratorios e Institutos de Salud Dr Carlos G Malbrán-ANLIS, Centro Nacional de Diagnóstico e Investigación en Endemo-Epidemias, Buenos Aires, Argentina

**Keywords:** phlebotomine sand flies, vectors, cytochrome c oxidase subunit 1 (COI)

## Abstract

**BACKGROUND:**

The *Nyssomyia* genus and *Lutzomyia* subgenus include medical important species that are Latin American leishmaniases vectors. Little is known about the phylogenetic relationships of closely-related species in each of these taxonomic groups that are morphologically indistinguishable or differentiated by very subtle details.

**OBJECTIVES:**

We inferred the phylogenetic relationships of closely-related species within both the *Nyssomyia* genus and the *Lutzomyia* subgenus using a cytochrome *c* oxidase subunit I (*COI*) fragment.

**METHODS:**

The sampling was carried out from 11 Argentinean localities. For genetic analyses, we used GenBank sequences in addition to our sequences from Argentina. Kimura 2-parameter (K2P) genetic distance and nucleotide divergence (*Da*) was calculated between closely-related species of *Nyssomyia* genus, *Lutzomyia* subgenus and between clades of *Lutzomyia longipalpis* complex.

**FINDINGS:**

The K2P and *Da* values within species of *Nyssomyia* genus and *Lutzomyia* subgenus were lower than the divergence detected between clades of *Lu. longipalpis* complex. The haplotype network analyses within *Lutzomyia* subgenus showed shared haplotypes between species, contrary to *Nyssomyia* genus with none haplotype shared. Bayesian inference within *Nyssomyia* genus presented structuring by species.

**MAIN CONCLUSIONS:**

This study evidences the phylogenetic proximity among closely-related species within *Nyssomyia* genus and *Lutzomyia* subgenus. The *COI* sequences of *Nyssomyia neivai* derived from the present study are the first available in GenBank.

Phlebotomine sand ﬂies (Diptera: Psychodidae) are involved in the transmission of arboviruses, bacteria and protozoans. Among these pathogens, the trypanosomatids of the genus *Leishmania* spp. Ross are the etiological agents of the leishmaniases, a zoonotic disease considered as neglected with special relevance in public health.^1^ Over 500 phlebotomine sand flies species distributed among 23 genera are known to occur in the Americas.^2^ Inside *Nyssomyia* genus, *Nyssomyia intermedia* (Lutz & Neiva), *Nyssomyia whitmani* (Antunes & Coutinho) and *Nyssomyia neivai* (Pinto) are *Leishmania braziliensis* Vianna vectors.^1^ ^,^ ^3^ *Nyssomyia whitmani* is also a *Leishmania shawi* Lainson, de Souza, Póvoa, Ishikawa & Silveira vector and is suspected to transmit *Leishmania guyanensis* Floch.^3^ ^,^ ^4^ *Nyssomyia intermedia* and *Ny. whitmani* are widely distributed in Brazil, including the five geographic regions of the country, but the latter is also registered in Suriname, French Guiana, Perú, Bolivia, Paraguay and northeast of Argentina. Whereas *Ny. neivai* is restricted to colder and drier areas at Bolivia, Paraguay, North, West-Central, Southeast and South regions of Brazil, and north of Argentina.^2^ ^,^ ^3^ Another medical significant species is *Lutzomyia longipalpis s.l.* (Lutz & Neiva), the most important neotropical vector of *Leishmania infantum* Nicolle, causative agent of visceral leishmaniasis.^5^ Its geographical distribution comprises a wide but discontinued range from central Mexico to Argentina and Uruguay, with a recently southern expansion and urban environments colonisation.^5^ ^,^ ^6^ A variety of evidence including chemical and behavioral suggests that it is a complex of at least seven species in Brazil, while molecular analyses identified eight haplogroups throughout its distribution.^5^ ^,^ ^7^ ^,^ ^8^ Regarding *Lutzomyia cruzi* (Mangabeira), its sibling, is also incriminated in Brazil as a vector of *Le. infantum* and suspected for transmitting *Leishmania amazonensis* Lainson & Shaw.^9^ While *Lutzomyia alencari* Martins, Souza & Falcão, has not been incriminated as a vector of *Leishmania* species.^3^ The geographic distribution of *Lu. cruzi* includes Bolivia and West-Central region of Brazil, while *Lu. alencari* is restricted to southeast of Brazil.^2^ ^,^ ^3^

Closely-related species but reproductively isolated that appear morphologically identical are often referred to as cryptic or sibling species, and a group of cryptic species is referred to as a species complex.^10^ The existence of sibling or closely-related species can hamper the process of species determination. Inside *Nyssomyia* genus, both males and females of *Ny. intermedia*, *Ny. whitmani* and *Ny. neivai* are closely-related species that can be determinate by subtle but sufficient morphological differences.^11^ On the other hand, inside *Lutzomyia* subgenus, the males of *Lu. longipalpis* complex, *Lu. alencari*, *Lu. cruzi, Lutzomyia pseudolongipalpis* Arrivillaga & Feliciangeli and *Lutzomyia matiasi* Le Pont & Mollinedo presents very subtle details for their differentiation, while the female of *Lu. longipalpis* complex, *Lu. cruzi* and *Lu. matiasi* are morphologically indistinguishable together with *Lutzomyia gaminarai* (Cordero, Vogelsang & Cossio).^11^ Therefore, females of these species have been identified based on their site of collection and through their association with males collected from the same location.^11^ Molecular and biochemical findings suggest that reproductive barriers do not prevent gene flow among them, and evidence of introgression has been reported between *Ny. intermedia* and *Ny. whitmani*, and between *Ny. neivai* and *Ny. intermedia*.^12^ ^,^ ^13^ Similarly, *Lu. longipalpis* complex and *Lu. cruzi* are not fully reproductively isolated with introgression occurring between them; the same is hypothesised for *Lu. longipalpis* complex and *Lu. alencari* in sympatric areas.^14^ ^,^ ^15^ ^,^ ^16^

Mitochondrial DNA (mtDNA) markers, as *COI* gene, have been used for phylogenetic relationships inference, phylogeography, population genetics and molecular taxonomic identification (DNA barcoding) in phlebotomine sand flies from the new and old world.^15^ ^,^ ^16^ ^,^ ^17^ ^,^ ^18^ ^,^ ^19^ Given the medical importance of some phlebotomine sand ﬂies within *Nyssomyia* genus and *Lutzomyia* subgenus, the studies of phylogenetic relationships will increase our understanding of the evolutionary relationships within these closely-related species, which to date has not yet been explored. Gene flow between these phlebotomine sand ﬂies may influence vector competence and capacity and could have important epidemiological consequences such as insecticide resistance or susceptibility to *Leishmania* infection.^13^ Therefore, the aim in the present study was to infer the phylogenetic relationships among closely-related phlebotomine sand flies within *Nyssomyia* genus (*Ny. intermedia*, *Ny. whitmani* and *Ny. neivai*) and within *Lutzomyia* subgenus (*Lu. longipalpis* complex, *Lu. cruzi* and *Lu. alencari*) using a *COI* fragment.

## MATERIALS AND METHODS

**Fig. 1:**
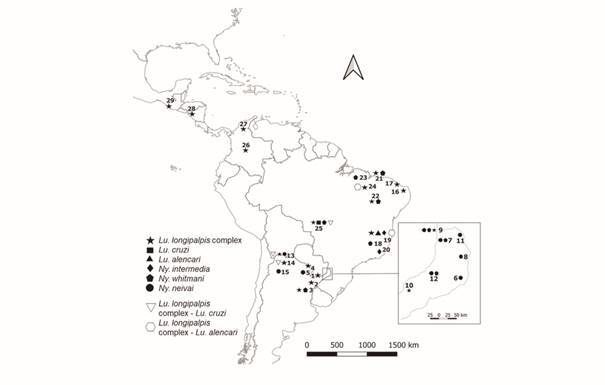
geographic distribution of *Lutzomyia longipalpis* complex (stars), *Lu. cruzi* (square), *Lu. alencari* (triangle), *Ny. intermedia* (diamond), *Ny. whitmani* (pentagon) and *Ny. neivai* (circle) specimens analysed. Haplotypes shared: H5 and H12 between *Lu. longipalpis* complex and *Lu. cruzi* (white inverted triangle). H59 between *Lu. longipalpis* complex and *Lu. alencari* (white hexagon). For more information on numbers and species in the map, revise Supplementary data (Table I).

*Study area, collection and taxonomic identification* - The Leishmaniasis Research Network in Argentina (REDILA) captured phlebotomine sand flies as part of different epidemiological projects in 11 localities from six Argentinean provinces: Corrientes, Entre Ríos, Formosa, Misiones, Salta and Tucumán during 2015-2018 [Fig. 1, Supplementary data (Table I)]. Phlebotomine sand flies captured using REDILA-BL ultraviolet light traps, were separated from other insects and were preserved in 90% ethanol until being processed. For identification purposes, the head and the last three segments were dissected individually, clarified and mounted on slides while the rest of the body was stored at -20ºC until DNA extraction. Species determination was based on morphological characters according to Galati´s taxonomic key, and genera abbreviations follow the nomenclature proposed by Marcondes.^11^ ^,^ ^20^ Reference specimens have been deposited at the Instituto Nacional de Medicina Tropical, Puerto Iguazú, Argentina.

*DNA extraction, ampliﬁcation and sequencing* - Genomic DNA was extracted individually from the rest of the body of the phlebotomine sand flies identified as *Lu. longipalpis* complex, *Ny. neivai*, *Ny. whitmani* and one *Migonemyia migonei* (França). We used DNA PuriPrep-S kit HighWay (INBIO, Argentina), following manufacturer’s instruction, with the addition of an incubation period at 56ºC that consisted in 4 h with vortex and spin every 20 min. The *COI* fragment amplification was done by a polymerase chain reaction (PCR) using the universal primers.^21^ The amplification conditions were an initial denaturalisation step of 94ºC for 4 min, followed by 35 cycles of 94ºC for 30 s, 56ºC for 30 s, 72ºC for 1 min, and a final extension of 72ºC for 10 min. PCR products were separated in 1.5% agarose gel electrophoresis, stained with 0.5 µL/mL SYBR Safe DNA (Invitrogen, USA) and isualized under blue light. The PCR products were purified with DNA PuriPrep-GP HW kit (INBIO, Argentina) following manufacturer’s instruction. The sequencing was carried out using both forward and reverse primers on an ABI 3730XLs (Macrogen, Korea) or was sent to the Canadian Centre for DNA Barcoding.

*Genetic analysis* - Cytochrome *c* oxidase subunit 1 (*COI*) sequences generated in this study of *Ny. whitmani*, *Ny. neivai* and *Lu. longipalpis* complex, (accession numbers in GenBank: MN857507-MN857540) in addition to sequences available from GenBank: *Ny. intermedia* (KP112715-KP112761), *Ny. whitmani* (KP112762-KP112770; MG234392-MG234412), *Lu. longipalpis* complex (JN845539- JN845542; KP112581-KP112595; KT806402-KT806405, KT806411, KT806413-KT806415, KT806417, KT806420, KT806426, KT806427, KT806430, KT806431, KT806437, KT806440, KT806443, KT806447, KT806451, KT806460, KT806468; GU909502-GU909505; HNLUZ004-HNLUZ005; MG234349-MG234370. BOLD Systems: MEXSM002-12.COI-5P, MEXSM003-12.COI-5P), *Lu. cruzi* (KP112572-KP112577) and *Lu. alencari* (KP112569-KP112571) were included in the analysis [Supplementary data (Table I)]. All sequences were manually aligned and edited using MEGA v.10 software. The multiple alignment of all sequences, was carried out in MEGA v.10 software. The number of polymorphic sites by species, the number of haplotypes and nucleotide divergence (*Da*) with the Jukes and Cantor (JC) correction among closely-related species were estimated using DnaSP v.6 software. Similarly, Kimura 2-parameter (K2P) genetic distances were estimated with 1000 replicates using MEGA v.10 software. The values of *Da* and K2P between *Lu. longipalpis* complex clades (see results) were also estimated.

Haplotype network of closely-related species was constructed using Network v.4.6.1.3, with the Median Joining algorithm to evaluate the relationships among haplotypes shared and the frequency distribution among species. The best-fitting model of evolution was estimated using the Bayesian Information Criterion (BIC) as implemented in JModeltest v.0.1.1 software. Bayesian inference (BI) was analysed with MrBayes v.3.2 software, four Markov Chain Monte Carlo (MCMC) were run for 10 million generations (sampled every 1000 generations) to allow adequate time for convergence (all ≤ 0.004369). The ﬁrst 25% of sampled trees were considered as burned in. The tree was visualised using FigTree v.1.4 (http://tree.bio.ed.ac.uk/software/ﬁgtree/). Sequences of *Mg. migonei* (access number: MT430890) and *Phlebotomus papatasi* Scopoli (access number: KY848828) were used as outgroups.

## RESULTS

Fifty-three phlebotomine sand flies were submitted to DNA extraction and *COI* amplification. Forty-eigth resulted in enough amplified product for sequencing (25 *Nyssomyia* genus and 23 *Lutzomyia* subgenus). Unfortunately, some of the sequences downloaded from GenBank and BOLD System were shorten than ours (658 bp). Then, during the multiple alignment all sequences were cut to the same length (543 bp for *Nyssomyia* genus and 483 bp for *Lutzomyia* subgenus).

*Nyssomyia genus: Ny. intermedia, Ny. whitmani and Ny. neivai* - A total of 103 *COI* sequences of 543 bp were analysed: 47 sequences of *Ny. intermedia*, 45 of *Ny. whitmani* and 11 of *Ny. neivai*. The species with more polymorphic sites was *Ny. whitmani* (44 sites, 8.1%). The values of K2P genetic distance ranged from 3.1 to 3.5%, while, the nucleotide divergence (*Da*) ranged from 0.017 to 0.025. The maximum and minimun K2P and *Da* values were between *Ny. intermedia* vs *Ny. neivai* and *Ny. whitmani* vs *Ny. neivai*, respectively (Table I).

**TABLE I:** Kimura 2-parameter (K2P) pairwise genetic distances between closely-related phlebotomine species within the *Nyssomyia* genus (below diagonal) and nucleotide divergence (*Da*) of the cytochrome *c* oxidase subunit I (*COI*) fragment measured as the number of net nucleotide substitution (above diagonal)

*Ny. intermedia*	*Ny. intermedia*	*Ny. whitmani*	*Ny. neivai*
		0.021 ± 0.001	0.025 ± 0.003
*Ny. whitmani*	0.034 ± 0.006		0.017 ± 0.002
*Ny. neivai*	0.035 ± 0.007	0.031 ± 0.006	

**Fig. 2:**
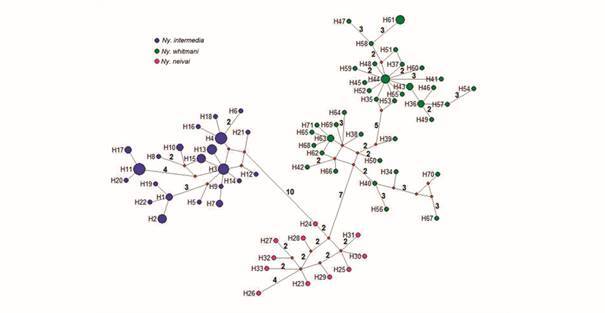
Median-joining haplotype network for *Nyssomyia intermedia*, *Ny. whitmani* and *Ny. neivai* based on 543 nucleotides of the cytochrome *c* oxidase subunit I (*COI*) gene. Haplotype frequency is represented by the size of nodes and missing haplotypes are shown as red circles. The line connecting haplotypes represents one mutational step, whereas numbers along the lines are total number of mutational steps. Colours indicate: blue = *Ny. intermedia*; green = *Ny. whitmani*; pink = *Ny. neivai*.

**Fig. 3:**
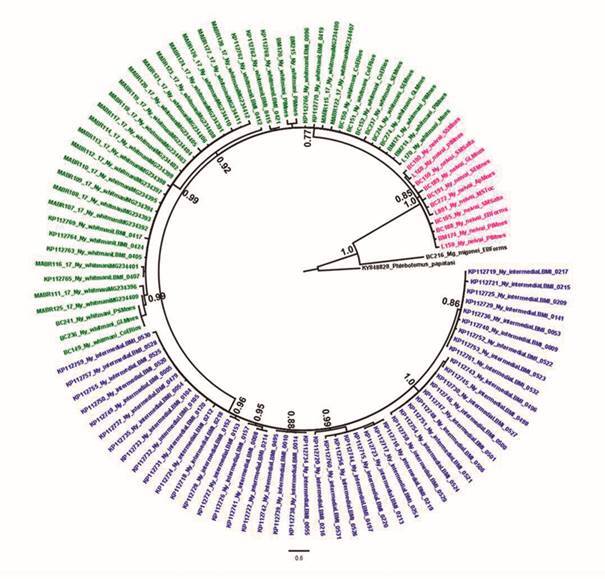
Bayesian inference (BI) topology tree for 543 nucleotides of the cytochrome *c* oxidase subunit I (*COI*) gene of *Nyssomyia intermedia*, *Ny. whitmani* and *Ny. neivai* inferred using the HKY+I+G model. Numbers on each Branch (above branch) represent posterior probabilities (PP) obtained in the BI. *Migonemyia migonei* and *Phlebotomus papatasi* were used as outgroup. The scale bar represents the expected number of nucleotide. Colours indicate: blue = *Ny. intermedia*; green = *Ny. whitmani*; pink = *Ny. neivai*.

Seventy-one haplotypes were identified from 103 *Nyssomyia* sequences, none haplotype was shared among them. Twenty-two haplotypes were identified in *Ny. intermedia* from localities of Brazil, 38 in *Ny. whitmani* from localities of Brazil and Argentina and 11 in *Ny. neivai* from Argentinean localities. The median-joining network identified at least 11 mutational steps between *Ny. intermedia* vs *Ny. neivai* and *Ny. whitmani* vs *Ny. neivai*, while between *Ny intermedia* vs *Ny. whitmani* there were at least 22 mutational steps. Argentinean *Ny. whitmani* (H61) from the northeast (Gobernador Lanusse and Piñalito Sur, Misiones provinces) and Pampeana (Concordia, Entre Ríos province) regions were identified by at least three mutational steps from Brazilian specimens (H58, Maranhão, Mato Grosso and Espírito Santo states) (Fig. 2). Bayesian inference (BI) was constructed using the HKY+I+G model as the most appropriate for the data (-lnL = 2152.4292; BIC = 5243.1277; Delta BIC = 0) with gamma of 0.1080 and p-inv of 0.6680. The analysis provided support with 0.77 posterior probability (PP) for the clade of *Ny. intermedia* and *Ny. whitmani*. *Nyssomyia intermedia* specimens grouped monophyletically with 0.96 PP, while some *Ny. whitmani* specimens also grouped monophyletically with 0.92 PP and another group presented polytomies (a comb-shaped phylogeny). Likewise, the basal clade with *Ny. neivai* specimens presented polytomies (Fig. 3).

*Lutzomyia subgenus: Lu. longipalpis complex, Lu. cruzi and Lu. alencari* - A total of 103 sequences of a 483 bp *COI* fragment were analysed: 94 sequences of *Lu. longipalpis* complex, six of *Lu. cruzi* and three *Lu. alencari*. Unfortunately, there were no sequences available in GenBank of *Lu. pseudolongipalpis*, *Lu. matiasi* and *Lu. gaminarai*, which are also closely-related species within the *Lutzomyia* subgenus.

*Lutzomyia longipalpis* complex showed the highest number of polymorphic sites (107 sites, 22%). The K2P genetic distance (ranged from 2.5 to 3.4%) and the nucleotide divergence (*Da*) (from 0.005 to 0.016) between closely-related species were low. The maximum and minimun K2P values were between *Lu. longipalpis* complex vs *Lu. cruzi* and *Lu. longipalpis* complex vs *Lu. alencari*, respectively. In contrast, the maximum and minimun *Da* values were between *Lu. cruzi* vs *Lu. alencari* and *Lu. longipalpis* complex vs *Lu. cruzi* and *Lu. alencari* (Table II). The intraspecific K2P distance and *Da* between clades from *Lu. longipalpis* complex (1 vs 2) (K2P = 9.8%, *Da* = 0.08) were high. Likewise, the values between clade 2 (samples from Colosó, Sucre) vs *Lu. cruzi* (K2P = 9.8%, *Da* = 0.081) and vs *Lu. alencari* (K2P = 9.6%, *Da* = 0.089) also were high [Supplementary data (Table II)].

**TABLE II:** Kimura 2-parameter (K2P) pairwise genetic distances between closely-related phlebotomine species within *Lutzomyi*a subgenus (below diagonal) and nucleotide divergence (*Da*) of the cytochrome *c* oxidase subunit I (*COI*) fragment measured as the number of net nucleotide substitution (above diagonal)

*Lu. longipalpis* complex	*Lu. longipalpis* complex	*Lu. cruzi*	*Lu. alencari*
		0.005 ± 0.003	0.005 ± 0.003
*Lu. cruzi*	0.034 ± 0.005		0.016 ± 0.009
*Lu. alencari*	0.025 ± 0.004	0.030 ± 0.006	

**Fig. 4:**
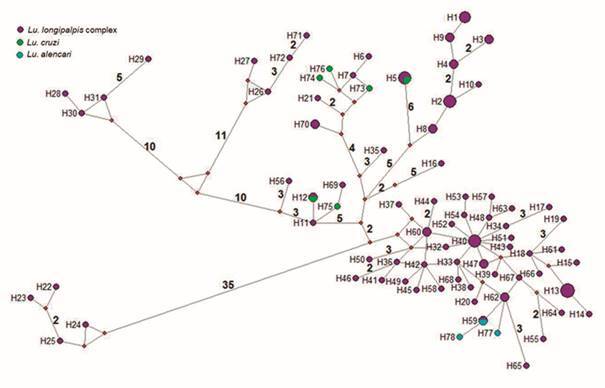
Median-joining haplotype network for the *Lutzomyia longipalpis* complex, *Lu. cruzi* and *Lu. alencari* based on 483 nucleotides of the cytochrome *c* oxidase subunit I (*COI*) gene. Haplotype frequency is represented by the size of nodes and missing haplotypes are shown as red circles. The line connecting haplotypes represents one mutational step, whereas numbers along the lines are total number of mutational steps. Colours indicate: purple = *Lu. longipalpis* complex; green = *Lu. cruzi*; turquoise = *Lu. alencari*.

**Fig. 5:**
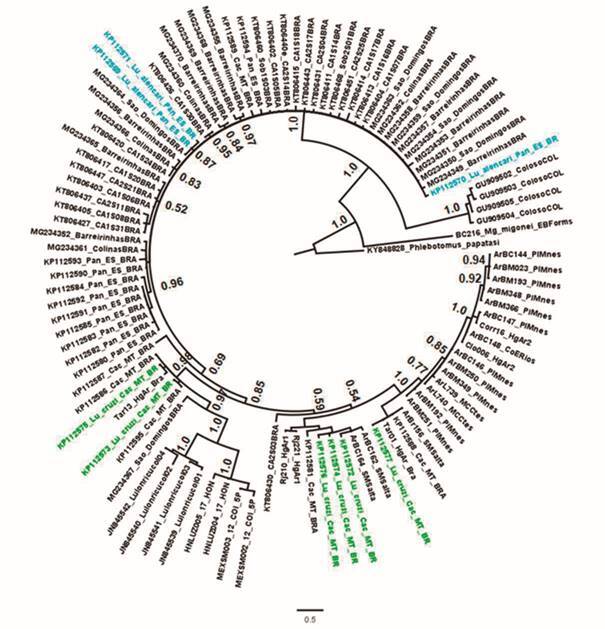
Bayesian inference (BI) topology tree for 483 nucleotides of the cytochrome *c* oxidase subunit I (*COI*) gene of *Lutzomyia longipalpis* complex, *Lu. cruzi* and *Lu. alencari* inferred using the HKY+G model. Numbers on each Branch (above branch) represent posterior probabilities (PP) obtained in the BI. *Migonemyia migonei* and *Phlebotomus papatasi* were used as outgroup. The scale bar represents the expected number of nucleotides. Colors indicate: black = *Lu. longipalpis* complex; green = *Lu. cruzi*; turquoise = *Lu. alencari*.

Seventy-eight haplotypes were identified from 103 sequences analysed of the three closely-related species of *Lutzomyia* subgenus: 72 haplotypes of *Lu. longipalpis* complex, six of *Lu. cruzi* and three of *Lu. alencari*. The minimum spanning haplotype network showed three haplotypes shared among species: H5 was shared between *Lu. longipalpis* complex (Mato Grosso, Brazil and Tartagal, Argentina) and *Lu. cruzi* (Mato Grosso, Brazil); H12 was shared between *Lu. longipalpis* complex and *Lu. cruzi* (both from Mato Grosso, Brazil); and H59 was shared between *Lu. longipalpis* complex (Maranhão, Brazil) and *Lu. alen*cari (Espírito Santo, Brazil). The specimens of *Lu. longipalpis* complex from Salta H6-H7, Tartagal H69 (Argentina) and Cáceres H11 (Brazil) are at least at one mutational step of *Lu. cruzi* H73 and H75 from Cáceres, Mato Grosso (Brazil). On the other hand, *Lu. longipalpis* complex specimens from Chiapas H71-H72 (Mexico) and Valle H26-H27 (Honduras) are separated by at least 24 and 26 mutational steps from Cundinamarca H28-H31 (Colombia, Andean region) and São Domingos do Maranhão H56 (Brazil) specimens, respectively (Fig. 4). Bayesian inference was constructed using the HKY+G model as the most appropriate for the data (-lnL = 2271.2317; BIC = 5852.6268; Delta BIC = 0) with gamma of 0.1040. The tree showed similar results to the network, its topology did not present structuration by species either by geography. Two monophyletic clades were identified: the first grouped specimens of *Lu. longipalpis* complex, *Lu. cruzi* and *Lu. alencari* (1.0 PP), while the second clade grouped only *Lu. longipalpis* complex specimens from Colosó, Colombia with 1.0 PP (Caribbean region) (Fig. 5). Indeed, *Lu. longipalpis* complex specimens from Colombia (Ricaurte, Cundinamarca and Colosó, Sucre) grouped within two monophyletic separated clades, the haplotype network identified at least 68 mutational steps between them (Figs 4-5).

## DISCUSSION

The findings of the present study evidence low values of both K2P genetic distance and nucleotide divergence (*Da*) between closely-related species within *Nyssomyia* genus and within *Lutzomyia* subgenus. The genetically closest species within *Nyssomya* genus were *Ny. whitmani* and *Ny. neivai*; while within *Lutzomyia* subgenus were *Lu. longipalpis* complex with *Lu. alencari* and with *Lu. cruzi*. Inside *Nyssomyia* genus, Mazzoni et al.^12^ ^,^ ^22^ reported lower divergence values than us between *Ny. intermedia* and *Ny. whitmani* from southeast Brazil using ten nuclear loci. This can be due to: (a) *Ny. neivai* specimens were not analysed, (b) differences in nucleotide substitution rate between nuclear and mitochondrial genes, (c) the number of samples analysed, (d) the smaller geographical area they assessed or (e) a combination among them. Currently, de Melo et al.^23^ reported even lower interspecific values inside *Bichromomyia flaviscutellata* complex between *Bichromomyia olmeca olmeca* (Vargas and Díaz-Nájera) and *B. olmeca bicolor* (Fairchild and Theodor). These authors concluded that these closely-related species represent the same species, supporting the morphological data. On the other hand, Scarpassa and Alencar,^18^ reported higher interspecific values (4.1-4.6%) between *Nyssomyia anduzei* (Rozeboom) and *Nyssomyia umbratilis* (Ward & Fraiha). Both are closely-related species with extensive overlapping areas and high morphological similarity. Arrivillaga et al.^17^ reported higher values of K2P distance ranging 1.3-17.7% within *Lu. longipalpis* complex while between *Lu. longipalpis* haplogroups (Hg) were 10.1-13%. In addition, K2P distance between Colombians specimens was comparable with the distance found between *Lu. longipalpis* complex clades (1 vs 2) in the present study. This could be explained by the geographical barriers in Colombia, such as the Andean Cordillera.^8^ ^,^ ^17^

The haplotype network and BI of *Nyssomyia* genus showed structuring by species although presented some polytomies, probably due to lack of samples. None haplotype was shared among species, contrary to mitochondrial introgression reported by Marcondes et al.^13^ between *Ny. intermedia* and *Ny. whitmani* as well as between *Ny. intermedia* and *Ny. neivai* using cyt *b* gene*.* Moreover, nuclear introgression using period gene was reported between *Ny. intermedia* and *Ny. whitmani* from southeast Brazil.^12^ ^,^ ^22^ In the present study, haplotype network separated these three closely-related species by various mutational steps. Indeed, *Ny. intermedia* and *Ny. whitmani* have 22 mutational steps between them. All *Ny. intermedia* specimens from Espírito Santo (Brazil) presented few mutational steps among haplotypes, may be due to the restricted geographical range covered. Similarly, Meneses et al.^24^ reported in *Ny. intermedia* population from Rio Janeiro (Brazil) few intra-population level polymorphism, using *COI*, 12S and 16S. In the BI, *Ny. whitmani* presented one monophyletic group (0.92 PP) and the rest of the specimens did not group (presenting polytomies). These not-grouped specimens from different localities of Brazil and Argentina are at least at seven mutational steps from the monophyletic group; suggesting that may form another monophyletic clade although more samples are needed to confirm this hypothesis. Indeed, haplotype network showed missing intermediate haplotypes between the two *Ny. whitmani* groups. Interestingly, Argentinian *Ny. whitmani* haplotypes from Concordia (its southernmost distribution until now) H61 and H62/H63 are separated by at least 17 mutational steps.^6^ Similarly, *Ny. whitmani* haplotypes from Puerto Iguazú (Northeast region, Argentina) have at least 11 mutational steps between them. These several mutational steps between the *Ny. whitmani* haplotypes from Argentina suggest that it could be a complex species occurring in sympatry. Ready et al.^4^ using cyt *b* gene suggested the presence of at least three phylogenetic lineages of *Ny. whitmani* in Brazil. Ishikawa et al.^25^ adding samples of *Ny. whitmani* from the State of Rondônia (Brazil), identified two clades, one from eastern Amazônia and one from the Atlantic forest zone of the northeast, although with low support value (< 50.0% bootstrap). Unfortunately, in the present study, *Ny. whitmani* specimens did not cover the same locations previously studied by Ready et al.^4^ and Ishikama et al.^25^ to compare the lineages. Regarding *Ny. neivai*, there are no studies available for comparisons, being these *COI* sequences the first available in GenBank.

*Lutzomyia* subgenus haplotype network and BI showed topologies without structuration by species neither by geography. The genetically closest species were *Lu. longipalpis* complex with *Lu. alencari* and with *Lu. cruzi*. *Lutzomyia longipalpis* complex shared haplotypes with both species or was found at least at one mutational step of them, compared with *Lu. cruzi* vs *Lu. alencari* that are separated by at least 16 mutational steps. Three haplotypes were shared among these species: (1) H5, *Lu. longipalpis* complex specimens from Argentina (Ar-Bra Hg) with *Lu. longipalpis* complex and *Lu. cruzi* from central-western Brazil, (2) H12, *Lu. longipalpis* complex with *Lu. cruzi* (both from central-western Brazil) and (3) H59, *Lu. longipalpis* complex (northeast Brazil) with *Lu. alencari* (southeast Brazil). In this sense, the shared haplotypes detected both in symphatry and allophatrycally may be explained by introgression among these species. The introgressive hybridisation seems to be a recurrent phenomenon that shaped the genome of *Lu. longipalpis* complex.^26^ This was suggested for *Lu. longipalpis* complex and *Lu. cruzi* based on molecular analyses using microsatellites markers^14^ and morphometrics analyses in sympatric areas.^27^

In addition, it was reported that the pheromone chemotype 1 (9MGB) was found in *Lu. cruzi* specimens (Bolivia and Brazil) and some *Lu. longipalpis* complex specimens from Honduras, Venezuela, Colombia, Brazil, Paraguay and Argentina.^7^ ^,^ ^28^ ^)^ Studies carried out by Pinto et al.^15^ and Rodrigues et al.^16^ on molecular taxonomic identification, supported the possibility of introgression events between *Lu. longipalpis* complex and both *Lu. cruzi* and *Lu. alencari.* In neighbour-joining analysis, they reported that *Lu. longipalpis* complex formed two groups, one included *Lu. cruzi* specimens from the Central-Western region, and the other grouped with *Lu. alencari* from Southeastern region (Brazil); however, they did not mention the existence of haplotypes shared among these closely-related species. Pinto et al.^15^ suggested that the clustering pattern is associated with the geographic distribution of *Lu. cruzi* and *Lu. alencari* and their sympatry with *Lu. longipalpis* complex.^3^ However, we detected two shared haplotypes approximately 1000 km apart (H5; H59). Concerning *Lu. longipalpis* complex itself, we included samples of the three genetic differentiated haplogroups (Ar1, Ar2, Ar-Bra) described in Argentina using ND4 and cyt *b* genes.^8^ We suggest that Ar2 could be the haplogroup that is spreading its distribution to southern Argentina given that Concordia’s specimen of *Lu. longipalpis* complex shared the H4 with Clorinda’s specimens. Concordia is located 800 km south of Clorinda and both localities belong to different ecoregions (Spinal and Humid Chaco, respectively). Furthermore, in BI Concordia’s specimen grouped with another specimen of Ar2 haplogroup also previously identified (1.0 PP). To test this hypothesis, more specimens and other genetic markers are needed.

The second monophyletic clade of *Lutzomyia* subgenus is represented only by Colombian specimens of *Lu. longipalpis* complex from Colosó, Sucre. The others Colombian specimens from Ricaurte, Cundinamarca clustered within clade 1, closer to specimens from Honduras and Mexico. Arrivillaga et al.^17^ reported specimens from Bucaramanga within the trans-Andean haplogroup and specimens from Bucaramanga and Neiva within the cis-Andean haplogroup using another *COI* gene fragment. Similarly, Pech-May et al.^8^ using ND4 and cyt *b* genes also found divergence in *Lu. longipalpis* complex from Colombia: Giron (Col1 Hg) vs El Callejón and Neiva (Col2 Hg) with at least 71 mutational steps between them, and a high genetic differentiation value (F_ST_ = 0.966) suggesting negligible gene exchange. In this study, the minimum spanning haplotype network showed that Colombian clades of *Lu. longipalpis* complex are separated by at least 68 mutational steps. This divergence in *Lu. longipalpis* complex from Colombia could be potentially due to vicariance, geographic and/or climatic barriers in addition to its low dispersal capacity.^8^ ^,^ ^17^ Contrary, the few genetic distance detected among closely-related species is probably related to evolutionary factors like incipient speciation and genetic introgression.^19^ The last one, as a consequence of natural hybridisation, might make the species to share traits of biomedical importance like vector competence, insecticide resistance, behavioral phenotypes and environmental adaptability, highlighting the need to focus on gene flow and the distribution of phenotypes of biomedical importance.^29^ Pinto et al.^30^ suggested that the phylogenetic proximity among *Lutzomyia* subgenus is congruent with their role as vectors of the same parasite; given that the physiologic mechanisms of these insects are likely to be similar due to recent common ancestry. Although *Lu. alencari*, *Lu. pseudolongipalpis* and *Lu. matiasi* have not been found naturally infected with *Leishmania*, attention should be given to their potential role in *Leishmania* transmission cycles, as these species are sister to incriminated vectors (*Lu. longipalpis* complex and *Lu. cruzi*).

This is the first study that evidence the phylogenetic proximity among closely-related phlebotomine species within *Nyssomyia* genus and within *Lutzomyia* subgenus. The relationships described here should be assessed using additional molecular markers (mitochondrial, nuclear, SNPs or microsatellites) with a broad sampling of all closely-related species, including to *Lu. pseudolongipalpis*, *Lu. gaminarai*, *Lu. matiasi*. In the case of *Lu. longipalpis* complex could be interesting to include all the haplogroups, given that the genetic distance seems to be higher within the complex that among the closely-related species. The presented results can be used as testable phylogenetic hypotheses and as a phylogenetic framework to future research using an integrative approach combining morphological, behavioral, chemical and multiple molecular markers with different evolution rate. Knowledge of phylogenetic relationships among relevant visceral and cutaneous leishmaniasis vectors and its siblings species (especially in sympatric areas) could be useful also in epidemiology control and strategies design, helping to assess if other species could be suspected of having a vectorial role in endemic leishmaniases areas.

## References

[1] Ready PD (2013). Biology of phlebotomine sand flies as vectors of disease
agents. Annu Rev Entomol.

[2] Shimabukuro PHF, De Andrade AJ, Galati EAB (2017). Checklist of American sand flies (Diptera, Psychodidae,
Phlebotominae) genera, species, and their distribution. Zookeys.

[3] Aguiar GM, Vieira VR, Rangel EF, Shaw JJ (2018). Regional distribution and habitats of Brazilian phlebotomine
species. Brazilian sand flies.

[4] Ready PD, Day JC, de Souza AA, Rangel EF, Davies CR (1997). Mitochondrial DNA characterization of populations of Lutzomyia
whitmani (Diptera Psychodidae) incriminated in the peri-domestic and
silvatic transmission of Leishmania species in Brazil. Bull Entomol Res.

[5] de Souza NA, Brazil RP, Araki AS (2017). The current status of the Lutzomyia longipalpis (Diptera
Psychodidae: Phlebotominae) species complex. Mem Inst Oswaldo Cruz.

[6] Santini MS, Manteca Acosta M, Utgés ME, Aldaz ME, Salomón OD (2018). Presence of Lutzomyia longipalpis and Nyssomyia whitmani in Entre
Rios, Argentina. Rev Inst Med Trop São Paulo.

[7] Araki AS, Vigoder FM, Bauzer LGSR, Ferreira GEM, Souza NA, Araújo IB (2009). Molecular and behavioral differentiation among Brazilian
populations of Lutzomyia longipalpis (Diptera Psychodidae:
Phlebotominae). PLoS Negl Trop Dis.

[8] Pech-May A, Ramsey J, González Ittig R, Giuliani M, Berrozpe P, Quintana M (2018). Genetic diversity, phylogeography and molecular clock of the
Lutzomyia longipalpis complex (Diptera Psychodidae). PLoS Negl Trop Dis.

[9] Falcão de Oliveira E.Oshiro ET.Fernandes WS.Ferreira AMT.Gutierrez
de Oliveira A.Galati EAB (2017). Vector competence of Lutzomyia cruzi naturally demonstrated for
Leishmania infantum and suspected for Leishmania amazonensis. Am J Trop Med Hyg.

[10] Black WC, Munstermann LE, Marquardt WC (2004). Molecular taxonomy and systematics of arthropods
vectors. Biology of disease vectors.

[11] Galati EAB (2018). Phebotominae (Diptera: Psychodidae): clasification, morphology
and terminology of adults and identification of American taxa. Morfologia e
terminologia de Phlebotominae (Diptera: Psychodidae). Springer.

[12] Mazzoni CJ, Araki AS, Ferreira GEM, Azevedo RVDM, Barbujani G, Peixoto AA (2008). Multilocus analysis of introgression between two sand fly vectors
of leishmaniasis. BMC Evol Biol.

[13] Marcondes CB, Day JC, Ready PD (1997). Introgression between Lutzomyia intermedia and both Lu neivai and
Lu. whitmani, and their roles as vectors of Leishmania
braziliensis. Trans R Soc Trop Med Hyg.

[14] Santos MFC, Ribolla PEM, Alonso DP, Andrade-Filho JD, Casaril AE, Ferreira AMT (2013). Genetic structure of Lutzomyia longipalpis populations in Mato
Grosso do Sul, Brazil, based on microsatellite Markers. PLoS One.

[15] Pinto I, Chagas BD, Rodrigues AAF, Ferreira AL, Rezende HR, Bruno RV (2015). DNA barcoding of neotropical sand flies (Diptera, Psychodidae,
Phlebotominae) species identification and discovery within
Brazil. PLoS One.

[16] Rodrigues BL, Carvalho-costa LF, Pinto IS, Manuel J, Rebêlo M (2018). DNA barcoding reveals hidden diversity of sand flies (Diptera
Psychodidae) at fine and broad spatial scales in Brazilian endemic regions
for leishmaniasis. J Med Entomol.

[17] Arrivillaga JC, Norris DE, Feliciangeli MD, Lanzaro GC (2002). Phylogeography of the neotropical sand fly Lutzomyia longipalpis
inferred from mitochondrial DNA sequences. Infect Genet Evol.

[18] Scarpassa VM, Alencar RB (2013). Molecular taxonomy of the two Leishmania vectors Lutzomyia
umbratilis and Lutzomyia anduzei (Diptera Psychodidae) from the Brazilian
Amazon. Parasit Vectors.

[19] Freitas M, Costa C, Sillva L, Leal-Balbino T, Balbino V (2016). Cytochrome oxidase I as tool to evaluate the Lutzomyia
longipalpis complex useful molecular marker or not?. Vector Biol J.

[20] Marcondes CB (2007). A proposal of generic and subgeneric abbreviations for
phlebotomine sandflies (Diptera Psychodidae: Phlebotominae) of the
world. Entomol News.

[21] Folmer O, Black M, Hoeh W, Lutz R, Vrijenhoek R (1994). DNA primers for amplification of mitochondrial cytochrome c
oxidase subunit I from diverse metazoan invertebrates. Mol Mar Biol Biotechnol.

[22] Mazzoni CJ, Souza NA, Andrade-Coelho C, Kyriacou CP, Peixoto AA (2006). Molecular polymorphism, differentiation and introgression in the
period gene between Lutzomyia intermedia and Lutzomyia
whitmani. BMC Evol Biol.

[23] de Melo LB, Alencar RB, Scarpassa VM (2020). Molecular taxonomy and phylogenetic inferences of Bichromomyia
flaviscutellata complex based on the COI gene DNA barcode
region. Infect Genet Evol.

[24] Meneses C, Cupolillo E, Monteiro F, Rangel E (2005). Micro-geographical variation among male populations of the
sandfly, Lutzomyia (Nyssomyia) intermedia, from an endemic area of American
cutaneous leishmaniasis in the State of Rio de Janeiro,
Brazil. Med Vet Entomol.

[25] Ishikawa EAY, Ready PD, de Souza AA, Day JC, Rangel EF, Davies CR (1999). Mitochondrial DNA phylogeny indicates close relationships between
populations of Lutzomyia whitmani (Diptera Psychodidae, Phlebotominae) from
the Rain-forest Regions of Amazônia and Northeast Brazil. Mem Inst Oswaldo Cruz.

[26] Araki AS, Ferreira GEM, Mazzoni CJ, Souza NA, Machado RC, Bruno RV (2013). Multilocus analysis of divergence and introgression in sympatric
and allopatric sibling species of the Lutzomyia longipalpis complex in
Brazil. PLoS Negl Trop Dis.

[27] Santos MFC, Andrade-Filho JD, Fernandes CES, Mateus NLF, Eguchi GU, Fernandes WD (2015). Morphometric analysis of Longipalpis (Diptera Psychodidae)
complex populations in Mato Grosso do Sul, Brazil. J Med Entomol.

[28] Spiegel CN, Dias DB, Araki AS, Hamilton JGC, Brazil RP, Jones TM (2016). The Lutzomyia longipalpis complex a brief natural history of
aggregation-sex pheromone communication. Parasit Vectors.

[29] Ready PD (2011). Should sand fly taxonomy predict vectorial and ecological
traits?. J Vector Ecol.

[30] Pinto I, Andrade J, Santos C, Falqueto A, Leite Y (2010). Phylogenetic relationships among species of Lutzomyia, subgenus
Lutzomyia (Diptera Psychodidae). J Med Entomol.

